# Identification of Genomic Regions for Partial Resistance to Soybean Rust Under Field Conditions Using FarmCPU and Machine Learning Approaches

**DOI:** 10.3390/plants15091385

**Published:** 2026-04-30

**Authors:** António Daniel Pedro Maquil, Tonny Obua, David L. Nsibo, Mildred Ochwo-Ssemakula, Harun Murithi, Paul Gibson, Ana Luísa Garcia-Oliveira, Richard Edema, Isaac Dramadri, Mohsen Yoosefzadeh-Najafabadi, Phinehas Tukamuhabwa

**Affiliations:** 1 Department of Crop Science and Horticulture, School of Agricultural Sciences, College of Agricultural and Environmental Sciences, Makerere University, Kampala P.O. Box 7062, Uganda; maquilantonio@yahoo.com.br (A.D.P.M.); mknossemakula@gmail.com (M.O.-S.); pgibson@agimprove.africa (P.G.); redema14@gmail.com (R.E.); onzigaisaac@gmail.com (I.D.); tphinehas@yahoo.com (P.T.); 2 Makerere University Center for Soybean Improvement and Development (MAKCSID), Makerere University, Kampala P.O. Box 7062, Uganda; 3 Faculdade de Engenharias, Universidade Pedagógica de Maputo, Campus de Ilhanguene, Av. Trabalho 2482, Maputo 1000-001, Mozambique; 4 Department of Plant and Soil Sciences, Forestry and Agricultural Biotechnology Institute (FABI), University of Pretoria, Pretoria 0028, South Africa; david.nsibo@fabi.up.ac.za; 5 International Institute of Tropical Agriculture (IITA), Nairobi 00100, Kenya; hmurithi@gmail.com; 6 Instituto Nacional de Investigação Agrária e Veterinária (INIAV), I.P., Estrada Gil Vaz, Ap. 6, 7350-901 Elvas, Portugal; 7 Makerere University Regional Centre for Crop Improvement (MaRCCI), College of Agricultural and Environmental Sciences, Makerere University, Kampala P.O. Box 7062, Uganda; 8 Department of Plant Agriculture, University of Guelph, Guelph, ON N1G 2W1, Canada; myoosefz@uoguelph.ca

**Keywords:** soybean rust, partial resistance, GWAS, machine learning, candidate gene

## Abstract

Soybean rust caused by the fungus *Phakopsora pachyrhizi* threatens global soybean production, causing yield losses of up to 80%. Race-specific *Rpp* genes provide short-term resistance due to pathogen variability, whereas partial resistance (PR) offers durable, broad-spectrum protection, though its genetic basis remains unclear. This study aimed to identify genomic regions and candidate genes underlying PR using the Fixed and Random Model Circulating Probability Unification (FarmCPU) genome-wide association study (GWAS) and machine learning (ML) methods, Random Forest (RF) and Support Vector Regression (SVR). A panel of 312 soybean accessions was evaluated under natural infection across six Ugandan environments. Rust index (RI), derived from rust severity and sporulation level, was used to estimate heritability (H^2^) and rank genotypes through Best Linear Unbiased Predictions (BLUPs), while Best Linear Unbiased Estimators (BLUEs) supported GWAS input. After quality control, 8272 SNPs were analyzed within a ±60 kb linkage disequilibrium (LD) window. Multi-environmental Analysis (MEA) of RI showed significant genetic effects (*p* < 0.01); H^2^ = 0.57–0.68. Sixty-one loci were detected: six by FarmCPU, 15 by RF, and 41 by SVR. Key genes included *Glyma.01G128100* (a WRKY transcription factor) and *Glyma. 13G228000*, receptor-like kinase) and *Glyma.20G173100* (WD40-domain regulator). Integrating ML with GWAS improved locus detection, confirming the polygenic nature of PR and supporting the use of genomic selection and locus pyramiding for durable rust resistance.

## 1. Introduction

Soybean rust (SBR), caused by the fungus *Phakopsora pachyrhizi,* represents one of the most destructive foliar diseases of soybean worldwide, causing yield losses that can exceed 80% when left uncontrolled [1]. The disease is particularly severe in tropical and subtropical regions, including Sub-Saharan Africa, where persistent humidity and extended leaf wetness create favorable conditions for pathogen infection and spread [2].

Fungicides remain the primary control strategy for SBR; however, this approach increases production costs, poses environmental risks, and promotes the emergence of fungicide-resistant pathogen populations [3]. Consequently, host-plant resistance constitutes the most sustainable and environmentally sound strategy for long-term SBR management [4].

To date, ten race-specific resistance loci (*Rpp1-Rpp7*, *Rpp6907*, *Rpp1-b*, *Rpp?*) have been identified and mapped to different soybean chromosomes [5,6,7]. These loci confer resistance to only specific pathogen races but frequently lose effectiveness due to the high virulence diversity and rapid evolution of *P. pachyrhizi* populations [4,8,9]. Although pyramiding multiple *Rpp* genes can extend resistance durability, its success depends on detailed knowledge of local pathotype composition, which remains largely unavailable in African breeding programs because of limited disease surveillance capacity [10,11].

As no single *Rpp* gene provides a broad-spectrum or durable resistance, breeding efforts have increasingly focused on partial resistance (PR), a quantitatively inherited trait governed by multiple loci with small individual effects [8,12,13]. PR reduces disease severity by limiting lesion development, delaying symptom progression and suppressing sporulation across environments [13,14,15]. Despite its importance for durable disease control, the genetic basis of PR remains poorly resolved [13,16]. Early bi-parental quantitative trait locus (QTL) mapping studies using Simple Sequence Repeat (SSR) microsatellite markers identified loci associated with PR to SBR [17,18], but this approach suffers from low marker density, limited allelic diversity, long population development times, and insufficient resolution for fine-scale genetic dissection [19].

Genotyping-by-sequencing (GBS) enables the cost-effective, genome-wide discovery of single-nucleotide polymorphisms (SNPs), thereby improving the resolution of genome-wide association studies (GWAS) for complex traits [20,21]. Unlike linkage mapping, GWAS exploits historical recombination and natural allelic diversity to detect marker-trait associations (MTAs), including small-effect loci contributing to PR [22,23]. These associations can subsequently support marker-assisted selection (MAS) for accelerated genetic improvement.

However, GWAS focusing on PR to SBR remains scarce. Most studies have relied on fixed SNP arrays (e.g., SoySNP50K) and conventional statistical models, including mixed linear models (MLM) and fixed and random model circulating probability unification (FarmCPU), using predominantly Asian, U.S., or Brazilian germplasm evaluated under controlled conditions [24,25,26,27,28,29]. In addition, some studies [29] analyzed genetically restricted panels, thereby limiting allelic diversity and constraining the discovery of novel PR loci. Conventional GWAS approaches also apply stringent significance thresholds and test SNPs individually, which reduces sensitivity to small-effect variants and overlooks epistatic interactions, contributing to the problem of missing heritability [16,30,31].

Recent advances in machine learning (ML), a subset of artificial intelligence (AI), provide powerful tools for modeling complex, non-linear and high-dimensional biological data [32]. Algorithms, such as Random Forest (RF) and Support Vector Regression (SVR), capture epistatic interactions and identify small-effect variants that often escape detection by conventional GWAS methods [33,34]. ML-integrated GWAS approaches have successfully identified stable loci for yield and seed-quality traits in soybeans [32,35]. This study evaluated a diverse soybean panel under natural field infection across multiple Ugandan environments and applied FarmCPU together with RF and SVR to prioritize genomic regions and candidate genes associated with PR to SBR.

## 2. Results

This section summarizes a multi-environment analysis aimed at quantifying phenotypic and genetic variation for partial resistance to soybean rust across maturity groups. Mixed-model approaches were used to estimate genotype performance and genotype-by-environment interactions, while genome-wide association analyses identified loci contributing to quantitative resistance.

### 2.1. Phenotyping

#### 2.1.1. Trait Variation for Rust Resistance Across Populations, Environments, Genotype Origins, Ranking

**Figure 1:**
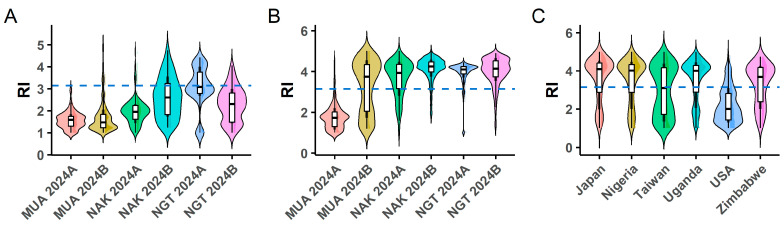
Distribution of rust index (RI) based on BLUEs across maturity groups (MG1: (**A**); MG2: (**B**)) and by origin (**C**). Panel C includes all 312 soybean genotypes grouped by country of origin. Resistant and susceptible reference varieties are included within their respective origin group and are not shown separately. The violin box plot summarizes the median line (interquartile range) and density.

Variation in the rust index (RI) was assessed across six environments in Uganda using a panel of 312 soybean genotypes. Genotypes were classified into two maturity groups: MG1 (80 genotypes), consisting exclusively of U.S. origin lines, and MG2 (232 genotypes), comprising lines from multiple geographic origins (Supplementary S1). Trait distribution was visualized using violin box plots based on BLUEs, showing the median, interquartile range, and density (Figure 1). Median RI values ranged from 1.5 to 4.8, with clear differences in spread and density across environments (Figure 1A,B). By origin, genotypes from Japan (4.2) consistently showed the highest values, whereas those from the USA (2.0) showed the lowest (Figure 1C). The six susceptible and seven resistant varieties were included within the 312-genotype panel as reference checks and were not treated as a separate group in origin-based analyses.

**Figure 2:**
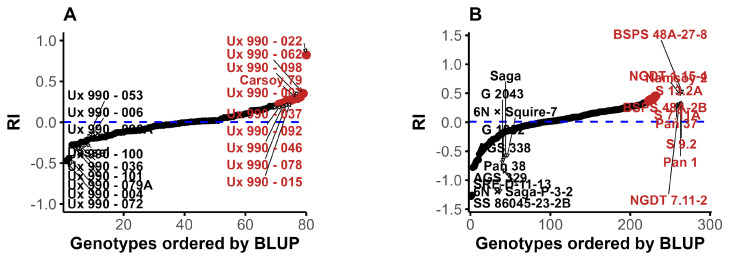
Ranking of genotypes for rust index (RI) based on BLUPs within two maturity groups. (**A**) MG1 (early maturing genotypes) and (**B**) MG2 (late maturing genotypes). In each panel, the x-axis shows genotypes ordered from lowest to highest BLUP values, and the y-axis shows the corresponding RI BLUPs. Negative BLUP values (black) indicate lower RI and higher partial resistance, whereas positive BLUP values (red) indicate higher RI and greater susceptibility. Selected genotypes at the resistance and susceptible extremes are labeled. The dashed blue horizontal line denotes the mean BLUP within each maturity group.

Genotype ranking was based on BLUP values from the multi-environment model, with the most resistant (black) and most susceptible (red) genotypes highlighted in Figure 2. In MG1, BLUPs ranged from −0.41 to 0.82, with Ux 990-072, Ux 990-004, and Ux 990-079A ranking highest for PR (Figure 2A). MG2 showed a broader range (−1.3 to 0.45), with SS86045-23-2B, AGS 329, and SRE-D-11-13 identified as top candidates (Figure 2B). Susceptible controls (Namsoy 2 and Namsoy 1 in MG2, and Williams 82 in MG1) clustered among the most susceptible genotypes (Figure 2A,B; Table S1). The two resistance checks, carrying resistance alleles at the *Rpp1* and *Rpp3* (UG5, harboring both loci), and Ankur (*Rpp3*), were associated with negative RI BLUP values (Table S3).

#### 2.1.2. Multi-Environment Analyses (MEA), Variance Component and Genetic Parameter in MG1 and MG2

The likelihood ratio test (LRT) from the MEA indicated that all model components were significant in MG2 with genotype effect (χ^2^ = 130.55; *p* < 0.01), environment (χ^2^ = 47.48; *p* < 0.01), and Genotype by environment (G × E) interaction effect (χ^2^ = 21.97; *p* < 0.01) (Table S3). In contrast, the G × E interaction was not significant in MG1 (χ^2^ = 1.72; *p* > 0.05) (Table S3).

**Figure 3:**
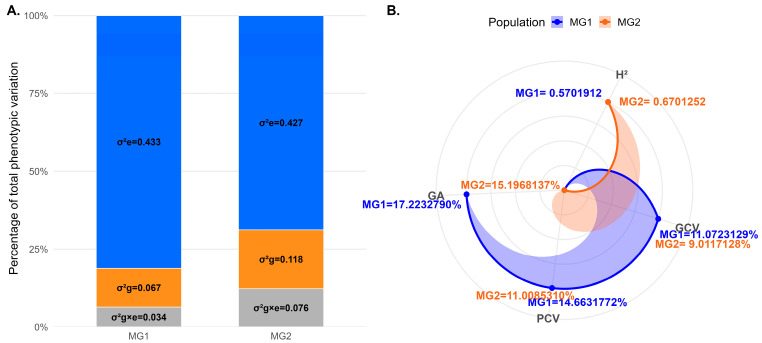
Variance component partitioning (**A**) and genetic parameter estimates (**B**) for two soybean populations. Residual variance ($\sigma_{\varepsilon }^{2}$, blue) represents the largest source of phenotypic variation, followed by genetic variance (σ^2^g, orange) and genotype × environment variance (σ^2^g × e, gray). Radar plots display genetic parameters for MG1 (blue) and MG2 (orange). Polygon elongation indicates the relative magnitude of each parameter: the sharp (blue) extension of MG1 toward genotype and phenotype coefficient of variation (GCV%, PCV%), and genetic advance (GA%) denotes high metric values, whereas the orange spike of MG2 toward broad-sense heritability (H^2^) indicates high heritability values.

The residual variance ($\sigma_{\varepsilon }^{2}$; blue) was the largest in both populations (0.43 in MG1 and 0.42 in MG2), while genetic variance (σ^2^_g_; orange) ranged from 0.07 to 0.12 and G × E variance (σ^2^_gxe_; gray) from 0.03 to 0.08 (Figure 3A). Broad-sense heritability (H^2^) ranged from 0.57 (MG1) to 0.67 (MG2) (Figure 3B). The genetic coefficient of variation (GCV%) varied between 9.01 and 11.07, and the phenotypic coefficient of variance (PCV%) between 11.01 and 14.66 (Figure 3B). Genetic advance as a percent of the mean (GA%) was 17.22% in MG1 and 15.20% in MG2 (Figure 3B).

#### 2.1.3. Genome-Wide SNP Landscape

A total of 17,300 SNPs were initially identified across the genome. After quality control filtering, 8272 high-quality SNPs were retained for downstream analyses, including GWAS and LD estimation.

**Figure 4:**
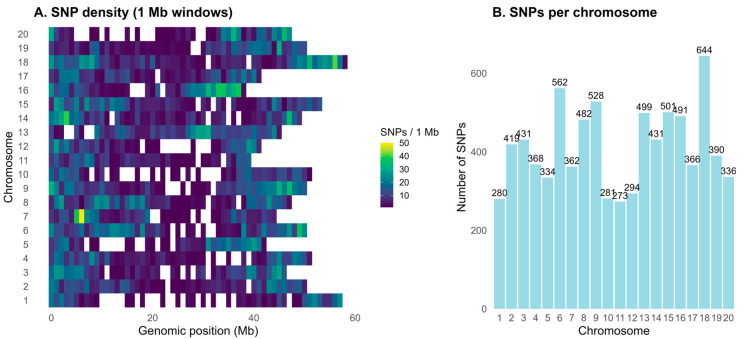
Genome-wide SNP landscape. (**A**) SNP density across chromosomes 1–20 in non-overlapping 1 Mb Windows. The color scale encodes the number of raw SNPs per window (white = 0, yellow = 50). (**B**) Total SNPs per chromosome (values above the bar).

The number of SNPs per chromosome ranged from 273 (Chr 11) to 644 (Chr 18) (Figure 4B). SNP density per 1 Mb window ranged from 0 to 50 SNPs/Mb (Figure 4A). The mean inter-marker distance varied from 77.6 kb (Chr 16) to 207.0 kb (Chr 1), with a genome-wide average of 124 kb (Table S4). The mean minor allele frequency (MAF) ranged from 0.221 (Chr 20) to 0.268 (Chr 15), with a genome-wide average of 0.246 (Table S4). Chromosome-level heterozygosity was below 0.8%, and the mean proportion of missing SNPs ranged from 0.003% to 0.13% (Table S4).

#### 2.1.4. Population Structure

**Figure 5:**
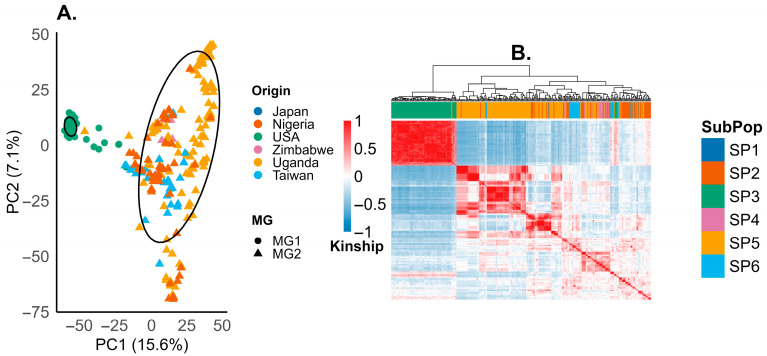
(**A**) Principal component analysis (PCA) of soybean accessions based on genome-wide markers. The x-axis reports principal component 1 (PC1, 15.6% of the total genetic variance), and the y-axis reports principal component 2 (PC2, 7.1% of the variance). Each point represents one accession, point color denotes geographic origin, and shapes denote maturity groups. The ellipse outlines the two main genetic clusters (MG1 and MG2). (**B**) genomic kinship matrix showing pairwise genetic relatedness among soybean accessions. Rows and columns correspond to accessions, ordered by hierarchical clustering, as shown in the dendrogram above the matrix. Cell color represents kinship coefficients, with red indicating higher relatedness and blue indicating lower relatedness. The color bar above the matrix assigns accessions to six genetic subpopulations (SP1–SP6).

Principal component analysis (PCA) resolved structure among the accessions (Figure 5A). The first two principal components explained 15.3% (PC1) and 7.4% (PC2) of the total variation. PC1 separated the panel into two main clusters by maturity group. All maturity group 1 (MG1) accessions (solid circles) clustered together and consisted exclusively of U.S. accessions (green), whereas maturity group 2 (MG2) accessions (triangles) formed a separate cluster and included accessions from Japan (dark blue), Taiwan (light blue), Uganda (yellow), Zimbabwe (red), and Pan-African varieties (orange).

The Kinship matrix identified six subpopulations (SP1–SP6) that corresponded closely to accessions origin and matched the PCA clustering pattern (Figure 5B).

**Figure 6:**
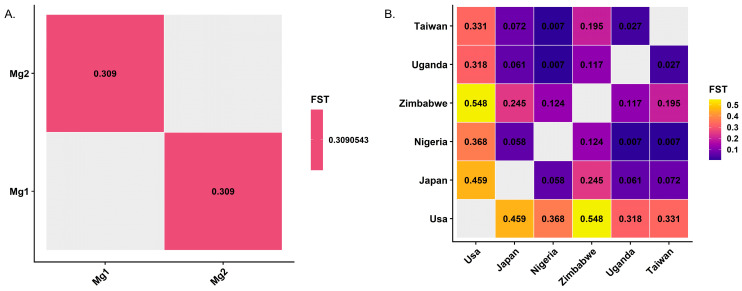
Genome-wide genetic differentiation (fixation index, FST) among soybean populations. (**A**) Mean FST between maturity groups MG1 and MG2 estimated from genome-wide SNP-allele frequencies (MG1-MG2 = 0.309). (**B**) Pairwise FST among geographic populations (USA, Japan, Nigeria, Zimbabwe, Uganda, Taiwan). Cell reports the pairwise estimate; the matrix is symmetric across the diagonal. Values range from 0.007 to 0.548. Color scale encodes FST magnitudes (lower = dark; higher = yellow).

Pairwise FST ranged from 0.007 to 0.548 (Figure 6A,B). The highest values were observed for USA-Zimbabwe (0.548) and USA-Japan (0.459), whereas the lowest were observed between Nigeria-Uganda (0.007) and Nigeria-Taiwan (0.007). The mean FST between MG1 and MG2 was 0.309 (Figure 6A).

Population structure estimates from PCA and Kinship analysis served as covariates in the FarmCPU GWAS model to reduce confounding effects.

#### 2.1.5. Linkage Disequilibrium (LD)

**Figure 7:**
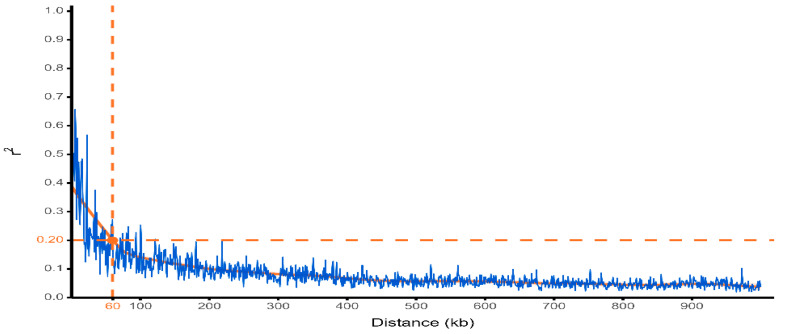
Linkage disequilibrium (LD) decay in 308 soybean genotypes. Pairwise r^2^ values decline with physical distance and cross r^2^ = 0.20 at ~60 kb.

LD decay was estimated in TASSEL 5 using the 8272 filtered SNPs based on pairwise r^2^. r^2^ declined below 0.20 at ~60 kb (Figure 7) and approached baseline levels (<0.10) beyond 200 k.

#### 2.1.6. Genome-Wide Association Analysis (GWAS)

**Figure 8:**
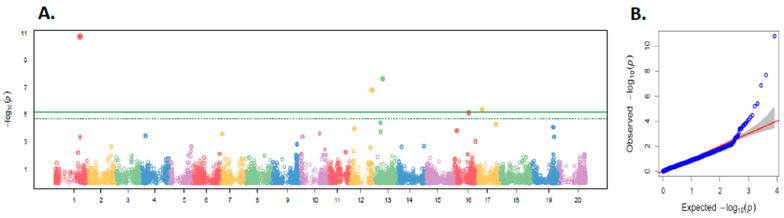
FarmCPU GWAS for rust index (RI). (**A**) Manhattan plot of −log 10(P) across chromosomes. (**B**) (QQ) plot of observed versus expected *p*-values, showing deviation from the diagonal red line at the upper tails (blue dots). The blue curve represents the patterns of LD decay, while the dashed orange horizontal and vertical lines indicate r = 0.20 and the corresponding physical distance (~60 kb), respectively.

**Figure 9:**
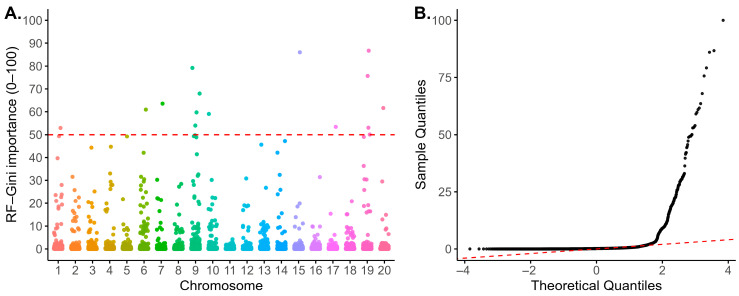
Random Forest GWAS results. (**A**) Manhattan plot of SNP importance score across chromosomes with the red dashed line indicating the empirical high-importance threshold. (**B**) QQ diagnostic plot comparing sample quantiles and theoretical quantiles of SNP importance.

**Figure 10:**
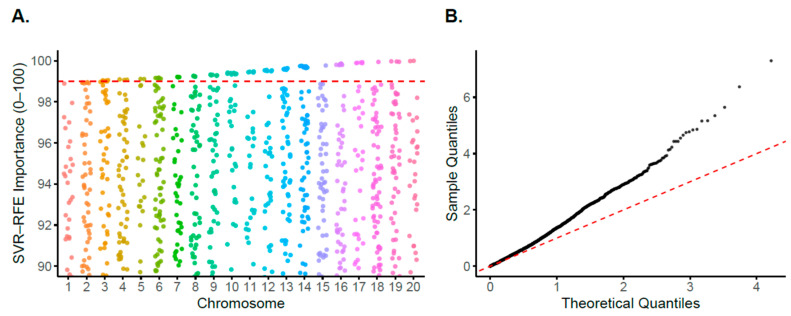
Support Vector Regression GWAS results. (**A**) Manhattan plot of SNP importance score across chromosomes, restricted 90–100 importance range. (**B**) QQ plot of SNP importance scores.

FarmCPU identified six SNPs, including a lead association on chromosome 1 (−log10P = 11) and additional loci on chromosomes 12, 13, 16 and 17 (Figure 8, Table 1 and Table S5). The Quantile-Quantile (QQ) plot is shown in Figure 8. RF detected 15 SNPs, distributed across chromosomes 1, 7, 9, 15, 19, and 20 (Figure 9, Table S5). SVR identified 41 SNPs, concentrated on chromosomes 2, 3, 7, 9, 10, 11, 12, 14, 16, 17, 19, and 20 (Figure 10, Table S5).

For RF and SVR, loci were prioritized using feature-importance scores above the 95th percentile. QQ plots are shown in Figure 9B and Figure 10B, respectively.

#### 2.1.7. Allelic Effects of Significant SNPs Associated with PR to SBR

Allelic effects of SNPs identified under the FarmCPU GWAS model were estimated as additive effects together with the proportion of phenotypic variance explained (PVE) (Table 1).

Additive effects ranged from −0.138 to 0.189. The locus ss14979891 (Chr 1: 45, 120, 978 bp) exhibited the largest effect (0.189) and accounted for 24.03% of phenotypic variance. The remaining loci showed additive effects between −0.138 and 0.091, with PVE values ranging from 0 to 2.231%.

**Figure 11:**
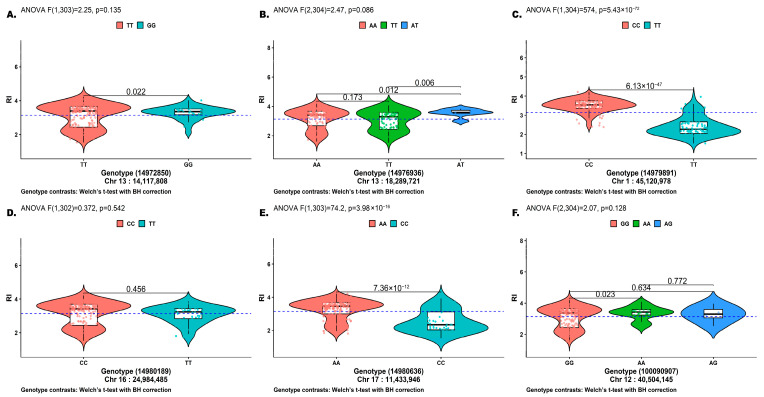
Genotype-specific phenotype distribution at FarmCPU identified loci for PR to SBR. Violin box plot shows RI values stratified by SNP genotype for loci identified under the FarmCPU GWAS model. Points represent individual observations. Central box plot indicates the median and interquartile range. The horizontal dashed line denotes the overall RI mean. Genotype effects were evaluated using one-way ANOVA(R version 4.5.2), with pairwise genotype contrasts assessed using Welch’s *t*-test with Benjamini–Hochberg correction. Corresponding F-statistic and *p* values are shown within each panel (**A**–**F**).

Genotype-specific phenotypic distributions are shown in Figure 11A–F. Significant genotype effects were detected for ss14979891 and ss14980636 (Figure 11C,E), with ANOVA results of F (1,304) = 574 (*p* = 5.43 × 10^−72^) and F (1,303) = 74.2 (*p* = 3.98 × 10^−16^), respectively. Pairwise contrasts (Welch’s *t*-test Benjamini–Hochberg correction) supported differences between CC and TT at ss14979891 (*p* = 6.13 × 10^−47^; TT lower mean RI), and between AA and CC at ss14980636 (*p* = 7.36 × 10^−12^; CC lower mean RI), respectively.

#### 2.1.8. Candidate Genes (GWAS + LD Window) and Functional Enrichment

Candidate genes were prioritized from LD—defined intervals (±60 kb) around lead SNPs and summarized by methods (Table 2). FarmCPU yielded six candidate genes, including the WRKY transcription factor (TF) (*Glyma.01G128100*) [36], an MLO-like membrane protein implicated in basal immunity, a gene involved in cell-wall biosynthesis (*RGP*), metal transport (*ATX1*) and transferase activity [37]. RF model prioritized several kinase-related genes (*Glyma.09G19700*, *Glyma.15G032850*), syntaxins, and leucine-rich repeat proteins (LRRs) linked to signal transduction and vesicle trafficking [38]. SVR recovered a broader repertoire dominated by receptor-like kinases (RLKs), nucleotide-binding leucine-rich repeat proteins (NLRs), including Toll/interleukin-1 receptor-nucleotide-binding site-leucine-rich repeat (TIR-NBS-LRR) immune receptors and transcription factors such as WRKY *(Glyma.10G138300*, *Glyma.12G097100*, *Glyma.14G028900*) and NAC family members [37]. In addition, the SVR candidate *Glyma.20G173100* encodes a WD40-repeat protein, supporting a role in post-translational modulation of immune signaling [38,39]. Cross-method overlap was limited to two genes, *Glyma.01G128100* (FarmCPU + RF) and *Glyma. 09G197000* (RF + SVR) (Table 2).

**Figure 12:**
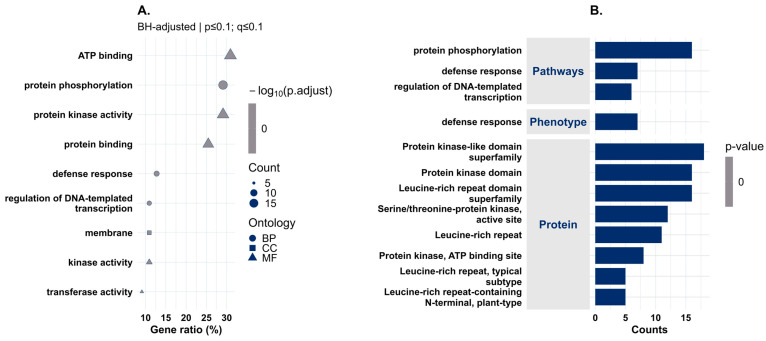
(**A**) GO enrichment analysis across biological process (BP), cellular component (CC), and molecular function (MF) categories.: The x-axis reports the gene ratio (%), point size represents the number of genes per GO term, and point shape denotes ontology category (circle = BP, square = CC, triangle = MF). Enrichment significance was evaluated using BH-adjusted *p*-values (*p* ≤ 0.1; q ≤ 0.1). After correction, only the MF term transferase activity remained significant (FDR = 0.091; n = 5). (**B**) Functional classification of candidate genes by enriched pathways, phenotypes and protein domains. Bar length represents the number of genes assigned to each category. This panel reports gene counts only and does not visually encode statistical significance.

Gene Ontology (GO) enrichment of SNP-mapped genes is shown in Figure 12 and Table S6. GO analysis highlighted molecular function (MF) and biological process (BP) categories related to protein phosphorylation, protein kinase activity and defense response (Figure 12A). After Benjamini–Hochberg correction (q ≤ 0.1), only the molecular function term transferase activity remained statistically significant (FDR = 0.091; *n* = 5; Table S6).

## 3. Discussion

The present study elucidates the genetic architecture of PR to SBR revealed through the integration of the FarmCPU GWAS model with the ML methods, RF, and SVR. This integrated GWAS-ML approach improves the detection of true association and reduces false positives compared with single-model analyses [32,33,34,35].

Using germplasm largely derived from the MARKSICD breeding program, this study revealed substantial phenotypic variation for soybean rust resistance (Figure 1 and Figure 2), confirming the quantitative nature of PR consistent with findings in Brazilian and Ethiopian germplasm [40,41,42]. Contrasting environmental conditions between locations strongly influenced disease expression (Figure 1; Table 3). At Nakabango, high humidity and prolonged leaf wetness favored *P. pachyrhizi* infection, whereas at Muarik, mainly in the first season, low rainfall, elevated temperature and reduced pressure limited pathogen development. These environmental contrasts account for the significant G × E interaction [2,43,44]. While field screening provides a realistic evaluation of resistance, climatic variability can hinder uniform disease establishment, highlighting the need for controlled-environment screening to complement field-based selection [45].

Environmental heterogeneity across sites and seasons (Table 3) was expected to modulate disease pressure, but our objective was to map PR loci with reproducible effects across contrasting field conditions. Accordingly, we first accounted for environmental and G × E effects in MEA mixed models and then performed GWAS on integrated BLUEs to enrich for stable, deployment-ready loci, thereby supporting breeders in selecting loci that remain effective across variable environments and pathogen pressures. Quantitative trait nucleotides (QTNs) by meteorological factor interaction frameworks [46,47] target phenotypic plasticity and environmental responsiveness, addressing distinct biological questions than durable, broad-environment PR.

Several accessions exhibited negative BLUP-RI values across environments (Figure 2), indicating consistent PR and corroborating previous findings [27,40,41,42]. These results underscore the global relevance of PR as a breeding target. Differences among genotype origins suggest historical selection under distinct pathotype pressures. A visual trend indicates that U.S. accessions tended to perform better than Japanese lines (Figure 1C), possibly due to canopy architecture that enhances solar penetration, and accelerates leaf surface drying, thereby reducing rust development [48]. Although the leaf area index (LAI) was not measured, evaluating the relationships between LAI and RI may clarify its potential as an indirect selection criterion. Integrating architectural and resistance traits can support the identification of genotypes with durable resistance to SBR [48].

The obtained heritability estimates (Figure 3B) were found to be moderate to high according to the classification previously described [49]. The results reported here were consistent with values reported for Brazilian panels (0.10–0.84) [40,41]. These findings suggest that PR expression is largely governed by genetic factors, with previous studies reporting 2 to 23 resistance loci [17], supporting its polygenic nature and potential for exploitation in breeding programs. This genetic complexity further underscores the suitability of GWAS as an effective approach for dissecting the underlying loci and identifying genomic regions associated with stable PR [50]. Principal Component Analysis (PCA) revealed two to six genetic clusters, linked to MG and origin (Figure 5), and consistent with other soybean panels [51]. This structure, together with cryptic kinship, requires strict correction in conventional GWAS to prevent false positives and biased effect estimates [27,28]. In rust resistance, the challenge is greater because applying minor allele frequency (MAF) thresholds (≥5%) reduces sensitivity to rare alleles, which may drive key variation and alter dominance across backgrounds and pathotypes [52,53]. Breeders should therefore re-estimate marker effects in local sub-panels, monitor the frequencies of rare alleles, and adjust genomic selection models to MG. Population-structure analyses also guide parent choice by identifying genetically complementary lines for recombination [54,55].

In this study, linkage disequilibrium (LD) decayed rapidly (~60 kb at r^2^ = 0.2), allowing for gene-level resolution (Figure 7). This decay is shorter than in cultivated soybean panels, 138 kb in the GmHapMap [56], 151 kb in landraces and 296 kb in other soybean germplasm, respectively [28,55], but longer than in wild *G. soja* (~2 kb at r^2^ = 0.2) and diverse global panels (~35–50 kb) [55,57]. This pattern reflects the intermediate recombination rate and genetic diversity of the MARKISCID panel, consistent with its mixed breeding background and supports accurate gene-level association mapping. Such variation highlights the dependence on population structure, germplasm background, mating system and sample size on LD decay [52]. Consequently, candidate gene mining was restricted to ±60 kb around each lead SNP, in line with the LD range observed here. Although haplotype or pangenome-based analyses could refine these intervals [56,58], the current resolution remains suitable for gene-level discovery.

The SVR and RF models identified numerous small-effect loci that were not detected by FarmCPU (Figure 8, Figure 9 and Figure 10), demonstrating that ML algorithms can capture non-linear associations and improve trait prediction [35]. These findings are consistent with the anticipated benefit of integrating an ML approach with conventional GWAS to enhance locus detection for PR by capturing additional small-effect variants contributing to complex trait variation (Table S5).

For breeding, this evidence highlights the importance of integrating multiple loci into selection pipelines rather than relying on single diagnostic SNPs. Incorporating multiple loci is particularly advantageous for managing the *P. pachirhizi* pathosystem under tropical conditions [2,4,59], especially in Sub-Saharan Africa, where rust surveillance remains limited [10].

Field-based GWAS addressing PR to SBR remain scarce, as most studies have focused on *Rpp*-mediated resistance [27]. To date, only one field-based GWAS has been conducted, identifying eight genome regions, six of which were novel [27]. Other GWAS have targeted different aspects of rust resistance [26,28,29]. In the present study, no direct overlap was detected with previously reported SNPs; however, several loci occurred near positions described in [27,28]. The lack of overlap likely reflects differences in SNP filtering criteria, the number of high-quality SNPs retained, or germplasm composition, consistent with findings from other soybean GWAS [57,60].

To facilitate biological interpretation of loci detected across the integrated GWAS-ML framework, Table 2 summarizes candidate genes identified by FarmCPU, RF, and SVR analyses, together with their associated GO terms, predicted function, and supporting literature.

**Table 2:** Candidate genes and predicted biological functions associated with partial resistance to soybean rust.

Methods	Candidate Genes	GO Terms	Functions	References
FarmCPU	*Glyma.01G128100*; *Glyma.13G083000*; *Glyma.12G230800*; *Glyma.17G141000*; *Glyma.16G111900*; *Glyma.13G053000*.	GO:0006952 (defense response); GO:0016021 (integral component of membrane); GO:0030001 (metal transport); GO:0046872 (metal binding)	Transcription regulation (WRKY), cell wall synthesis (RGP), metal binding (ATX1), membrane-related defense (MLO), Protein modification (O-fucosyltransferase), unknown (UDF641)	[36,37,61]
RF	*Glyma.19G019100*; *Glyma.15G032850*; *Glyma.09G197000*; *Glyma.07G156000*; *Glyma.20G229500*; *Glyma.09G206800*; *Glyma.09G014900*; *Glyma.19G019900*; *Glyma.19G171100*; *Glyma.01G128100*; *Glyma.01G135600*	GO. 0003993 (inositol-phosphate kinase); GO:0004674 (protein serine/threonine kinase activity); GO:0005484 (SNAP receptor activity), GO:0005515 (protein binding), GO:0006886 (intracellular protein transport), GO:0016020 (membrane), GO:0016192 (vesicle-mediated transport)	Signal transduction, transcription regulation (WRKY-like), membrane trafficking (syntaxin), kinase-mediated defense (RLKs)	[38,62,63]
SVR	*Glyma.20G173100*; *Glyma.19G108800*; *Glyma.19G051200*; *Glyma.17G232235*; *Glyma.17G030000*; *Glyma.17G018800*; *Glyma.16G210800*; *Glyma.16G200600*; *Glyma.16G185200*; *Glyma.16G182751*; *Glyma.16G156100*; *Glyma.14G199400*; *Glyma.14G173900*; *Glyma.14G114700*; *Glyma.14G080100*; *Glyma.14G060400*; *Glyma.14G043300*; *Glyma.14G028900*; *Glyma.14G017200*; *Glyma.13G373000*; *Glyma.13G310500*; *Glyma.13G228000*; *Glyma.13G166100*; *Glyma.12G212500*; *Glyma.12G191200*; *Glyma.12G097100*; *Glyma.12G091200*; *Glyma.12G088900*; *Glyma.11G147500*; *Glyma.11G098000*; *Glyma.11G063100*; *Glyma.10G221200*; *Glyma.10G138300*; *Glyma.10G110100*; *Glyma.10G032000*; *Glyma.09G197000*; *Glyma.08G017400*; *Glyma.07G191900*; *Glyma.03G048100*; *Glyma.02G189400*	GO:0005515 (protein binding); GO:0006952 (defense response); GO:0009607 (response to biotic stimulus); GO:0007165 (signal transduction), GO:0043531 (ADP binding); GO:0003700 (DNA binding TF activity), GO:0043565 (DNA binding); GO:0004674 (protein ser/thr kinase activity	Kinase-mediated signaling, transcription regulation (WRKY, NAC), membrane stress response (MLO, ERD), defense-related proteins (NLR/TIR-NBS-LRR) and post-translational regulation via WD40-repeat proteins.	[37,38,39,64,65]

**Table 3:** Monthly agrometeorological parameters at each trial site across the season (2024A and 2024B).

Environment	TMIN (°C)	TMAX (°C)	TM (°C)	RH (%)	PP (mm)
MUARIK 2024A	19.6	26.8	23.2	77.2	116.075
MUARIK 2024B	18.35	26	22.213	79.75	273.35
NAKABANGO 2024A	20.375	26.575	23.5	79.275	136.5
NAKABANGO 2024B	19.3	25.675	22.475	78.85	154.075
NGETTA2024A	20.375	31.65	26.025	78.78	126.2
NGETTA2024B	18.2	27.85	23.025	79.05	200.275

TMIN, minimum air temperature; TMAX, maximum air temperature; TM, mean air temperature; RH, relative humidity; PP, total precipitation.

A stable SNP on chromosome 1 detected by both FarmCPU and RF (Figure 8 and Figure 9 and Table 2), co-located with *Glyma.01G128100* (*GmWRKY4*) [36], a WRKY transcription factor regulating phenolic biosynthesis and callose deposition [37,61,66]. This locus likely enhances antimicrobial metabolite accumulation and represents a strong candidate for MAS. Additional WRKY-like loci on chromosomes 10, 12 and 14 (Table 2) indicate a distributed WRKY-regulatory network that reinforces quantitative defense signaling across the genome [36,37]. Slicing of *GmWRKY* genes has been associated with increased susceptibility to *P. pachyrhizi*, whereas resistance genotypes show rapid WRKY induction after infection [67]. These results extend previous WRKY-based defense models in soybean [68], positioning *GmWRKY4* and its homologs as central transcriptional nodes that integrate multiple small-effect loci to sustain durable, quantitative resistance.

The convergence of FarmCPU and SVR (Table 2) detected loci within the region surround in on *GmLMM1* (*Glyma.13G054400)* highlights this area as a promising target for further investigation. Given that *GmLMM1* encodes a receptor-like kinase regulating pattern-triggered immunity (PTI) and is exploited by multiple pathogens [62,63], it represents a strong candidate for functional validation in the context of resistance to *P. pachyrhizi*.

In addition to kinase-mediated signaling, the SVR model identified a WD40-domain locus Glyma. *20G173100* on chromosome 20, encoding a WD repeat-containing protein 5-like (Table 2). WD40/DWD proteins act as substrate receptors in the CUL4-DDB1 ubiquitin ligase complex and regulate defense-related factors. In soybean, for example, DWD proteins (including *Gm08DWD* interacts with R1-type MYB factor *GmMYB176* to modulate isoflavonoid biosynthesis [65], and in sugar beet, *BvWD40-82* enhances stress tolerance [64], underscoring the defense/stress relevance of this class. To our knowledge, *Glyma.20G173100* has not been functionally characterized for defense in soybean, making it a novel candidate for investigating ubiquitin-mediated metabolic reprogramming underlying PR to *P. pachyrhizi*.

No significant association was found on chromosome 18, a region that hosts several *Rpp*-mediated resistance genes, including Rpp6907-7/Rpp690-4 [8,69]. This likely reflects germplasm differences, pathotype diversity, and the predominance of PR over single-gene resistance in this panel, consistent with the view that field-based PR is polygenic and driven by multiple small-effect loci [14].

Despite these advances, this study has several limitations. Phenotypic evaluation relied on natural infection rather than controlled inoculation, which likely increased environmental variance and reduced resolution for isolate-specific effects. At the genomic level, the moderate marker density (~8272 k SNPs), constrained fine-mapping resolution at some loci, and the use of the Wm82.a4 reference genome may have excluded alleles unique to African germplasm. Under these constraints, this study prioritized candidate genes using an integrative GWAS-based framework that combined association strength, LD structure, physical proximity to lead SNPs, functional annotation, GO enrichment and support from homologous genes reported in the literature, rather than direct functional validation.

Gene expression is a context-dependent molecular phenotype whose interpretation requires appropriate biological and experimental alignment. In the absence of transcriptomic data generated for the GWAS-evaluated genotypes, expression-based analyses would not provide reliable validation of identified loci. Moreover, GWAS is designed to prioritize candidate genes based on statistical association and LD structure rather than transcript abundance. Accordingly, this study focused on gene-level discovery and statistical characterization of allelic effects. Functional validation of candidate alleles will require fine-scale genotyping, haplotype dissection, and controlled experimental assays. Transcriptomic analyses are, therefore, more appropriately addressed in downstream functional investigations.

Future studies should integrate pangenome or multi-reference assemblies to improve variant discovery and refine LD boundaries, followed by high-density sequencing, transcriptome profiling, haplotype-based analyses, and controlled inoculation experiments to validate key loci such as *Glyma. 01G128100* (*WRKY*), Gm *LMM1* and *Glyma.20G173100* (*WD40*). Building on validated loci, genomic selection (GS) strategies that capture the cumulative effects of multiple small-effect variants, together with the development of Kompetitive Allele-Specific Polymerase Chain Reaction (KASP) markers, will enable targeted pyramiding with *Rpp* genes to achieve durable, broad-spectrum resistance and reduce reliance on fungicides.

## 4. Conclusions

This study demonstrated that PR to SBR under field conditions is governed by a polygenic architecture that can be effectively resolved by integrating multi-locus statistical GWAS (FarmCPU) with machine-learning-based association analyses (RF and SVR). The combined analytical framework consistently identified genomic regions associated with quantitative resistance, supporting the robustness of loci detected across methods.

Key resistance-associated regions, including those harboring Glyma, 01G128100 (GmWRKY4), GmLMM1, and Glyma.20G173100, implicate transcriptional regulation, receptor-like kinase signaling, and ubiquitin-mediated post-translational control as central components of the PR response to *Phakopsora pachyrhizi*. These results indicate that durable field resistance arises from coordinated regulatory and signaling pathways rather than from single major resistance genes.

By linking statistically supported genomic regions with biologically relevant defense mechanisms, this work provides a mechanistic basis for interpreting quantitative resistance in soybean. The loci identified here represent prioritized candidate regions for downstream functional validation and provide a genomic framework for marker-assisted breeding strategies aimed at stabilizing resistance across environments.

## 5. Materials and Methods

### 5.1. Plant Material

A panel of 312 soybean (*Glycine max*) accessions was evaluated for partial resistance (PR) to soybean rust (SBR). The panel was obtained from the Makerere University Center for Soybean Improvement and Development (MAKCSID), Uganda, and comprised materials from Uganda (140), the United States (80), Taiwan (27), Japan (19), Zimbabwe (13), and Nigeria (33, Pan African Variety Collection). The panel included 13 released Ugandan varieties, 122 advanced breeding lines, 87 landraces, and 90 accessions of unknown improvement status. To monitor disease pressure and possible pathotype variability, six susceptible and seven resistant checks were included, of which two carried known *Rpp* genes (*Rpp1* and *Rpp3*). These reference varieties were included in the full 312-genotype panel and were not treated as a separate category. Full panel details are provided in Table S1.

### 5.2. Experimental Locations and Season Description

Field trials were conducted in three SBR hotspots, namely Makerere University Agricultural Research Institute Kabanyolo (MUARIK), Nakabango, and Ngetta ZARDI, across two consecutive seasons in 2024 (2024A and 2024B) in Uganda. These locations are known for their high natural inoculum pressure [70]. Monthly agrometeorological data for each location and season were retrieved from the NASA POWER database (https://power.larc.nasa.gov/) using the nasapower R package (version 4.5.2) [71] for the crop cycle from emergence to R6 growth stage (March–June for 2024A; August–December for 2024B) (Table 3).

### 5.3. Experimental Design and Field Management

**Figure 13:**
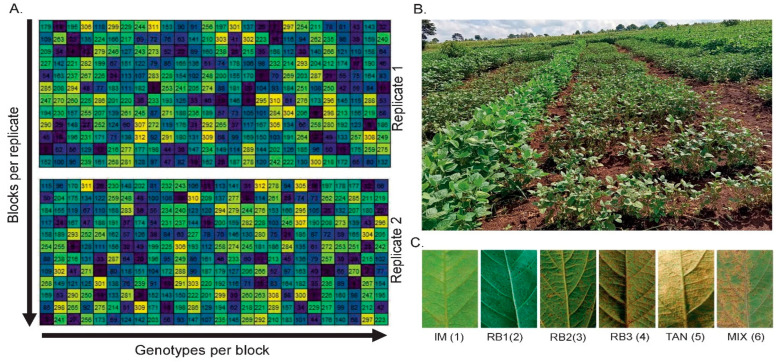
Field layout and lesion-type scoring were used to evaluate soybean rust resistance. (**A**) Alpha lattice design illustrating the spatial distribution of 312 soybean genotypes across two replications, each comprising 12 incomplete blocks with 26 plots. Each colour code represents a soybean genotype within the experiemntal layout. (**B**) Soybean field trial under natural infection by a *P. pachyrhizi* population. (**C**) Lesion type scale used for classification: Immune (IM = 1), reddish-brown without sporulation (RB1 = 2), minimal sporulation (RB2 = 3), moderate sporulation (RB3 = 4), tan lesion with abundant sporulation (TAN = 5), and mixed lesion types (MIX = 6).

The trials followed a 12 × 26 alpha lattice design with two replications per environment (location × season). Randomization was performed using the *agricolae* package in R 4.4.2 [72]. Each 2.4 m^2^ plot consisted of two 2 m rows spaced 0.6 m apart with 0.05 m between plants. Rows of susceptible check, ‘Namsoy 2’, were sown perpendicular to the test plots to enhance disease spread (Figure 13A,B). No fertilizers were applied. Caterpillar infestation was managed by foliar spraying with lambda-cyhalothrin (50 g L^−1^ a.i.; applied at 2 mL L^−1^, Jubaili Aggrotech) 34 days after planting, using a 15 L knapsack sprayer with a 1.2 mm nozzle. Weeding was done manually at different soybean growing and reproductive stages.

### 5.4. Phenotyping

Rust symptoms were monitored from the first appearance at 3–5 time points per environment. Five permanently marked plants per plot were sampled, and the most symptomatic leaflet from the mid-canopy trifoliate was scored. Rust severity (RS) was scored on a 1–9 scale using area diagrams [73]. Sporulation level (SL) was rated on a 1–5 scale [74]. To integrate both traits, RS was rescaled to a 1–5 scale (Y=0.5X+0.5), and the rust index (RI) was computed as: $RI=\sqrt{\left(RS\times SL\right)}$. Low RI values indicated minimal symptoms, while higher values reflected a more severe infection [75]. Only the final assessments conducted at the R6 growth stage were considered, as they represent the peak expression of disease and more apparent genotype differentiation.

### 5.5. DNA Extraction and Genotyping-by-Sequencing

Young trifoliate leaves were collected two weeks after germination during seed multiplication in the second planting season of 2023 (July–December 2023) at Makerere University, Agricultural Research Institute, Kabanyolo (MUARIK), Uganda. Genotypes were sown at 60 × 5 cm spacing to ensure uniform growth. Leaf samples were freeze-dried for 72 h using a Savant MODULVO D Thermoquest system (Savant Instruments, Holbrook, NY, USA). Genomic DNA was extracted using the Nucleomag Plant Kit (Macherey-Nagel GmbH & Co.KG, Duren, Germany), yielding 50–100 ng/μL [76]. DNA integrity was verified on a 0.8% agarose gel. Genotyping was conducted by SEQART Africa (Nairobi, Kenya) using the DArTseq platform (Diversity Arrays Technology, Canberra, Australia) as described in [77]. Briefly, genomic DNA was digested with *PstI* and *MseI*, ligated to barcoded and standard adapters, and PCR-amplified. Sequencing libraries were generated and run on the Illumina HiSeq 2500 system with 77-base single-end reads. Marker calling was performed using DArTsoft 14, with both marker types scored as binary (1 = presence, 0 = absence). Markers were aligned to the *Glycine max* reference genome (Wm82.a4.v1) to assign chromosomal position [77,78].

### 5.6. Statistical Analysis

#### 5.6.1. Phenotype Data Analysis

Genotypes were classified into two maturity groups (MGs) based on observed days to maturity: MG1 (early, 70–84 days; 80 genotypes) and MG2 (late, 85–114 days; 232 genotypes), together constituting the full panel (312 genotypes). Phenotypic data were analyzed using linear mixed models fitted by restricted maximum likelihood (REML) to account for experimental design effects, environmental variation and genotype x environment interaction. Analyses were conducted in R 4.4.2 [79] using the *lme4* package [80] and followed a two-stage framework consisting of single-environment analyses (SEA) and multi-environment analyses (MEA). All analyses were performed separately by maturity group to minimize confounding from phenological differences.

Data normality and variance homogeneity of variances were evaluated using the Shapiro–Wilk and Levene’s test, respectively (Table S2), prior to mixed-model fitting.

##### Stage 1: Single-Environment Analysis (SEA)

Each alpha-lattice trial was analyzed independently using the linear model:

(1) $$Y_{ijk}=\mu +G_{i}+R_{j}+B_{k(j)}+\varepsilon_{ijk}$$

where Y_ijk_ represents the observed value for the i-th genotype in the j-th replication and k-th block nested within j-th replication; μ is the overall mean; G_i_ denotes the fixed effect of the i-th genotype; R_j_ is the fixed effect of the j-th replication; B_k(j)_ represents the random effect of the k-th block nested within the j-th replication, (B_k(j)_∼Niid (0, σ^2^_b_)), and ϵ_ijk_ is the residual error term, (ϵ_ijk_∼Niid (0, σ^2^ε)).

Traits repeatability (r^2^), and coefficient of variation (CV%) were calculated as:

(2) $$R-squaredr^{2}=\frac{\sigma_{g}^{2}}{\sigma_{g}^{2}+\frac{\sigma_{\varepsilon }^{2}}{n_{r}}}$$

(3) $$CV\left(\%\right)=(\frac{\sigma_{\varepsilon }}{\bar{Y}})*100$$

where r^2^ represents repeatability, σ^2^_g_ is the genetic variance, $\sigma_{\varepsilon }^{2}$ is the residual variance, and *nr* is the number of replications, $\sigma_{\varepsilon }$ standard deviation and $\bar{Y}$ is the grand means.

Environments failing at least two of the following: r^2^ < 0.3, CV ≥ 30% or non-significant genetic effect (*p* > 0.05), were excluded, as MUA 2024A (Table S2). Best linear unbiased estimates (BLUEs) from the retained environments served as the basis for the multi-environment analysis (MEA).

##### Stage 2: Multi-Environment Analysis (MEA)

A combined model across environments was fitted as:

(4) $$Y_{ijklm}=\mu +G_{i}+E_{j}+(G{\times E)}_{ij}+\varepsilon_{ijklm}$$

where Y_ijklm_ is the observed value for the i-th genotype in the j-th environment, k-th replication nested within the j-th environment, and the l-th block nested within the k-th replication, and the j-th environment; μ is the overall mean; G_i_ is the random effect of the i-th genotype (G_i_~N_iid_ (0, σ^2^_g_)); E_j_ is a random effect of the j-th environment, defined by combination of location and season, (E_j_~N_iid_ (0, σ^2^_e_)); (G × E)_ij_ is the random interaction effect between the i-th genotype and the j-th environment, ((G × E)_ij_~N_iid_ (0, σ^2^_ge_)); and ε_ijklm_ is residual error, assumed to be independently and identically distributed (ϵ_ijkl_~N_iid_ (0, σ^2^ε)).

From this model, BLUEs were obtained by fitting genotypes as fixed effects and were used as phenotypic input for GWAS to avoid double shrinkage [81]. When genotypes were treated as random effects, the same model produced BLUPs, which provide shrinkage-adjusted predictions of genotype performance by integrating information from all environments through the mixed-model structure and accounting for genotype-by-environment interactions and data imbalance. BLUPs were used for genotype ranking (Figure 2) and for estimation of genetic parameters (Figure 3). Violin box plots (Figure 1) were produced in R [79] using the *ggplot2* [82], *ggdist* and *patchwork* packages to illustrate the distribution of the RI derived from multi-environment BLUEs across maturity groups (MG1 and MG2) and genotypic origins.

Model inferences followed standard mixed model procedures. Fixed effects were evaluated using the Wald test, whereas random effects were assessed using the likelihood ratio test (LRTs) based on the REML fitted model. For each random effect, inference was obtained by comparing a full model including the variance with a reduced model excluding that component, with the test statistic calculated as −2 times the differences in REML Log-likelihoods and evaluated against a chi-square [80].

##### Variance Component and Genetic Parameter Estimation

Variance components and genetic parameters were estimated from the multi-environment mixed model fitted with genotypes as random effects and visualized in bar and radar plots [83].

##### Broad-Sense Heritability

Heritability was estimated based on variance components from the random effects model following [44]:

(5) $$H^{2}=\frac{\sigma_{g}^{2}}{\sigma_{p}^{2}}=\frac{\sigma_{g}^{2}}{\sigma_{g}^{2}+\frac{\sigma_{g*e}^{2}}{n_{e}}+\frac{\sigma_{\varepsilon }^{2}}{n_{e}*n_{r}}}$$

where *σ^2^_g_* is the genetic variance; $\sigma_{p}^{2}$ is the total phenotypic variance; σ^2^_g*e_ is the genotype environment interaction variance. $\sigma_{\varepsilon }^{2}$ is the residual variance; ***n_e_*** is the number of environments, and *n_r_* is the number of replications.

Genotypic (GCV) and phenotypic (PCV%) coefficients of variation, along with genetic advance as a percentage of mean (GA%), were estimated according to [49]:

(6) $$PCV(\%)=\frac{\sqrt{\sigma_{p}^{2}}}{\bar{X}}*100$$

(7) $$GCV(\%)=\frac{\sqrt{\sigma_{g}^{2}}}{\bar{X}}*100$$

(8) $$GA(\%)=\frac{H^{2}}{\bar{X}}*k\sigma_{P}*100$$

In Equation (8), $\sigma_{P}$ is the standard of phenotypic deviation, and k = 2.06 is the selection intensity at 5% selection pressure under a normal distribution population; $\bar{X}$ is the trait mean.

##### Pre-Processing of Genotypic Data

Genotypic data were pre-processed in TASSEL 5 [84]. The complete panel comprised 312 genotypes, all of which were retained for phenotypic evaluation and multi-environmental analyses.

For GWAS, additional genotypic quality control and biological filtering were applied. SNPs with a minor allele frequency (MAF) < 0.05, >20% missing data, or >20% heterozygosity were removed, and missing values were imputed using a nearest-neighbors algorithm [30]. Given the panel size and the level of missingness typical of reduced-representation genotyping platforms (GBS/DArT), a conservative MAF threshold of ≥0.05 was used to ensure reliable allele-frequency estimation and to reduce spurious associations driven by rare alleles.

Two genotypes were excluded due to poor SNP quality, and two additional genotypes carrying known *Rpp* resistance loci were intentionally removed to avoid confounding major gene effects with partial resistance. The final GWAS dataset, therefore, comprised 308 genotypes and 8272 high-quality SNPs. Marker density was evaluated relative to the linkage disequilibrium (LD) structure of the panel. LD decay was estimated empirically, with r^2^ declining to 0.2 at approximately ±60 kb (Figure 7). Under the observed LD structure, the retained SNP set provides a genome-wide set suitable for association mapping of loci with stable effects [85]. This LD extent was used to assess genome-wide marker coverage and to define the window for candidate gene identification.

##### Analysis of Population Structure

Population structure was assessed using principal component analysis (PCA) based on SNP data. Taxa names in genotypic and phenotypic datasets were harmonized using Unicode-safe normalization with the *stringi R* package [86]. Genotypic data were numerically encoded (AA = 0, AG/GA = 1, GG = 2), and monomorphic markers were removed. The standardized genotypic matrix was analyzed using the *prcomp ( )* function in R [79]. The first two principal components (PC1 and PC2) were visualized with the ggplot2 package [82], color-coded by genotype origin.

Pairwise FST was estimated using the Weir and Cockerham estimator implemented in *hierfstat* R package [87].

##### Genome-Wide Association Study (GWAS)

GWAS was conducted using the Fixed and Random Model Circulating Probability Unification (FarmCPU) model [88] as the primary mixed-model framework for association mapping. To complement the parametric test, two widely used machine learning (ML)-based approaches, Random Forest (RF) [89] and Support Vector Regression (SVR) [90], were applied to capture non-linear marker-trait relationships and distributed small-effect loci characteristic of complex traits [31]. Association signals detected consistently across FarmCPU and ML-based methods were considered robust due to their convergence approaches. Analyses were performed using multi-environment BLUEs to identify loci with stable effects across environments.

**FarmCPU**

FarmCPU was implemented in GAPIT [88]. Population structure and relatedness were controlled using the first three principal components (PC1–PC3) and a TASSEL-derived kinship matrix (centered identity by states methods), included as covariates in GAPIT. The model iteratively fits a random and fixed effects model.

**The random-effect model was:**

(9) $$Y_{I}=u_{I}+\varepsilon_{I}$$

where Y_i_ is the phenotype values of the i-th individual, $u_{I}$ is the total genetic effect, and e_i_ is the residual effect.

**The fixed effect model was:**

(10) $$Y_{i}=\sum_{t=1}^{T}N_{it}F_{t}+M_{it}K_{J}+\varepsilon_{i}$$

where Y_i_ is the phenotype values of the i-th individual, N_it_ represents the genotype values of the t-th pseudo-QTNs; Ft is its fixed effect; M_ij_ represents the genotype value of the j-th SNP, K_j_ its effect, and e_i_ is the residual.

Marker significance was determined using both false discovery rates (FDR) and the Bonferroni correction. SNPs were considered significant at FDR < 0.05 or at a Bonferroni-adjusted threshold of *p* < 0.05/n, corresponding to *p* < 6.04 × 10^−6^. Allelic effects of significant SNPs were visualized using violin box plots of phenotype by genotype. Genotype effects were tested by one-way ANOVA with Welch-adjusted pairwise contrasts and Benjamini–Hochberg correction.

**Random Forest (RF)**

RF [89] was applied as an ensemble regression method that aggregates multiple decision trees, where the final prediction was obtained by averaging across the outputs of the individual trees, as expressed in Equation (11).

(11) $$Y_{i}=\frac{1}{B}\sum_{b=1}^{B}T_{b}\left(X_{I}\right)$$

where Y_i_ represents the predicted value for genotype Xi, Tb is the total number of built trees, and B is the total number of trees; a total of 1000 trees (B) were generated using bootstrapped samples.

**Support Vector Regression (SVR)**

SVR [90] modeled the SNP-phenotype relationship by minimizing an ε-insensitive loss function, which ignores errors within ε and penalizes deviations beyond this margin. Using kernel functions, the model maps SNP input vectors into high-dimensional feature spaces, enabling both linear and nonlinear regression. The regression function is expressed as:

(12) Y=W·φ(c)+b

where Y represents the phenotypic values, W is the weight vector orthogonal to the hyperplane, φ(c) is a nonlinear transformation of the SNP vector c, and b is the intercept. The ε-insensitive margin constrains prediction within Y=W·φ(c)+b±ε.

##### Implementation and Evaluation of Machine Learning-Based GWAS Model

RF and SVR were implemented in R (version 4.4.2) [79] using the *caret* package [91]. RF was executed via the *ranger* package [92] using impurity-based importance (Gini impurity criterion). SVR was implemented with a linear kernel, and SNP selection was ranked using recursive feature elimination (RFE), which interactively removed the least informative SNPs based on model performance. To ensure robustness and minimize overfitting, a 5-fold cross-validation scheme with 10 repetitions was applied to the training dataset [32]. The importance score of SNPs from both methods was rescaled to a 0–100 scale, with 100 indicating the highest importance. A global empirical threshold of 5% to retain informative SNPs, and for visualization, SVR Manhattan plots were zoomed to the 90–100 range to highlight the most predictive markers (Figure 10A).

Model performance (Figure S1) was evaluated by (i) Mean Squared Error (MSE), (ii) Root Mean Squared Error (RMSE), (iii) Mean Absolute Error (MAE), (iv) Mean Absolute Percentage Error (MAPE), (v) Ratio of performance to deviation (RPD), and (vi) Coefficient of Determination (R^2^), following the equation [93,94]:

(13) $$MSE=\frac{1}{n}\sum_{i=1}^{n}e_{i}^{2}$$

(14) $$RMSE=\sqrt{\frac{\sum_{i=1}^{n}e_{i}^{2}}{n}}$$

(15) $$MAE=\frac{1}{n}\sum_{i=1}^{n}{|e}_{i}|$$

(16) $$MAPE=\frac{100}{n}\sum_{i}^{n}\frac{{|e}_{i}|}{{|y}_{i}|}$$

(17) $$RPD=\frac{SD}{RMSE}$$

(18) $$and\;R^{2}=1-\frac{{SS}_{residual}}{{SS}_{total}};\;\;{SS}_{total}=\sum_{j=1}^{n}{\left(y_{i}-\bar{y}\right)}^{2}\;and\;{SS}_{residual}=\sum_{i=1}^{n}e_{i}^{2}$$

where $e_{i}^{2}=y_{i}-{\hat{y}}_{i}$ and $y_{i}$ are the observed and $\hat{y}$ predicted values, respectively, and $\bar{y}$ grand mean. The standard deviation (SD) of the observed values was calculated as:

(19) $$SD=\sqrt{\frac{1}{n-1}\sum_{i=1}^{n}{\left(y_{i}-\bar{y}\right)}^{2}}$$

##### Candidate Gene Mining and Functional Enrichment from GWAS Methods

Candidate genes were prioritized and identified by GWAS and machine learning-approaches using LD-defined intervals (±60 kb at r^2^ = 0.2; Figure 7), estimated for the study population. Genomic coordinates were mapped to the *Glycine max* Williams 82 reference genome (Wm82.a4.v1) using SoyBase [95]. Gene model (glyma.Wm82.gnm4.ann1.T8TQ.gene_models_main.gff3.gz) was imported into R via the rtracklayer package [96], restricted to annotated “gene” and “pseudogene” features, standardized to Glyma identifiers and merged with functional annotations (glyma.Wm82.gnm4.ann1.T8TQ.info_gene_annot.txt.gz).

Prioritization relied on physical proximity to lead SNP within LD intervals, and gene functional annotation. Gene Ontology (GO) and InterPro enrichment analyses were conducted using the *clusterProfiler* 4.0 package [97], with annotations retrieved from the *biomaRt* package [98]. Significance was determined using Benjamini–Hochberg correction (*p* ≤ 0.1; q ≤ 0.1) and a minimum gene set size of five (minGSSize = 5), with all SNP-mapped genes used as the background.

The study design targeted association-based locus discovery and annotation-supported gene prioritization. Gene expression profiling, differential expression analysis, haplotype-based fine mapping or experimental functional validation were not included. RNA sequencing (RNA-seq) or targeted quantitative reverse transcription polymerase chain reaction (qRT-PCR) datasets generated from the same GWAS-evaluated genotype under comparable experimental conditions were unavailable.

## Figures and Tables

**Table 1:** Significant SNPs associated with PR to SBR identified by FarmCPU. Negative SNP effect values indicate alleles associated with reduced phenotypic values of PR to SBR, whereas positive values indicate alleles associated with increased phenotypic values under the fitted additive model.

SNP ID (ss)	Chr ^a^	Pos ^b^	*p*-Value	Allele	Effect	PVE (%) ^c^
14979891	1	45,120,978	1.71234 × 10^−11^	C/T	0.18906918	24.0345233
14976936	13	18,289,721	2.15314 × 10^−8^	T/A	−0.08086888	2.30802392
100090907	12	40,504,145	1.42323 × 10^−7^	G/A	−0.137702623	0
14980636	17	11,433,946	4.06185 × 10^−6^	C/A	0.086254818	2.16663329
14980189	16	24,984,485	6.75758 × 10^−6^	T/C	0.091080129	0.01117974
14972850	13	14,117,808	3.6107 × 10^−5^	G/T	−0.089186901	0.04977956

**^a^** Chromosome; **^b^** Physical position (bp), **^c^** Phenotypic variance explained (PVE, %).

## Data Availability

The original contributions presented in this study are included in the article/Supplementary Material. Further inquiries can be directed to the corresponding author.

## Supplementary Materials

The following supporting information can be downloaded at: https://www.mdpi.com/article/10.3390/plants15091385/s1, Table S1–S6: Candidate gene lists, functions, keywords classification, and enrichment results.

## Abbreviations

The following abbreviations are used in this manuscript:

PR	Partial resistance
SBR	Soybean rust
MG	Maturity group
MAS	Marker assistant selection
ML	Machine learning
LD	Linkage disequilibrium
SNP	Single-nucleotide polymorphism
GWAS	Genome-wide association study
GS	Genome selection
