# Supplementary figures and images for: Identification of Genomic Regions for Partial Resistance to Soybean Rust Under Field Conditions Using FarmCPU and Machine Learning Approaches
Source: Plants (Basel). 2026 Apr 30;15(9):1385. doi: 10.3390/plants15091385 (PMC13164846; doi:10.3390/plants15091385)

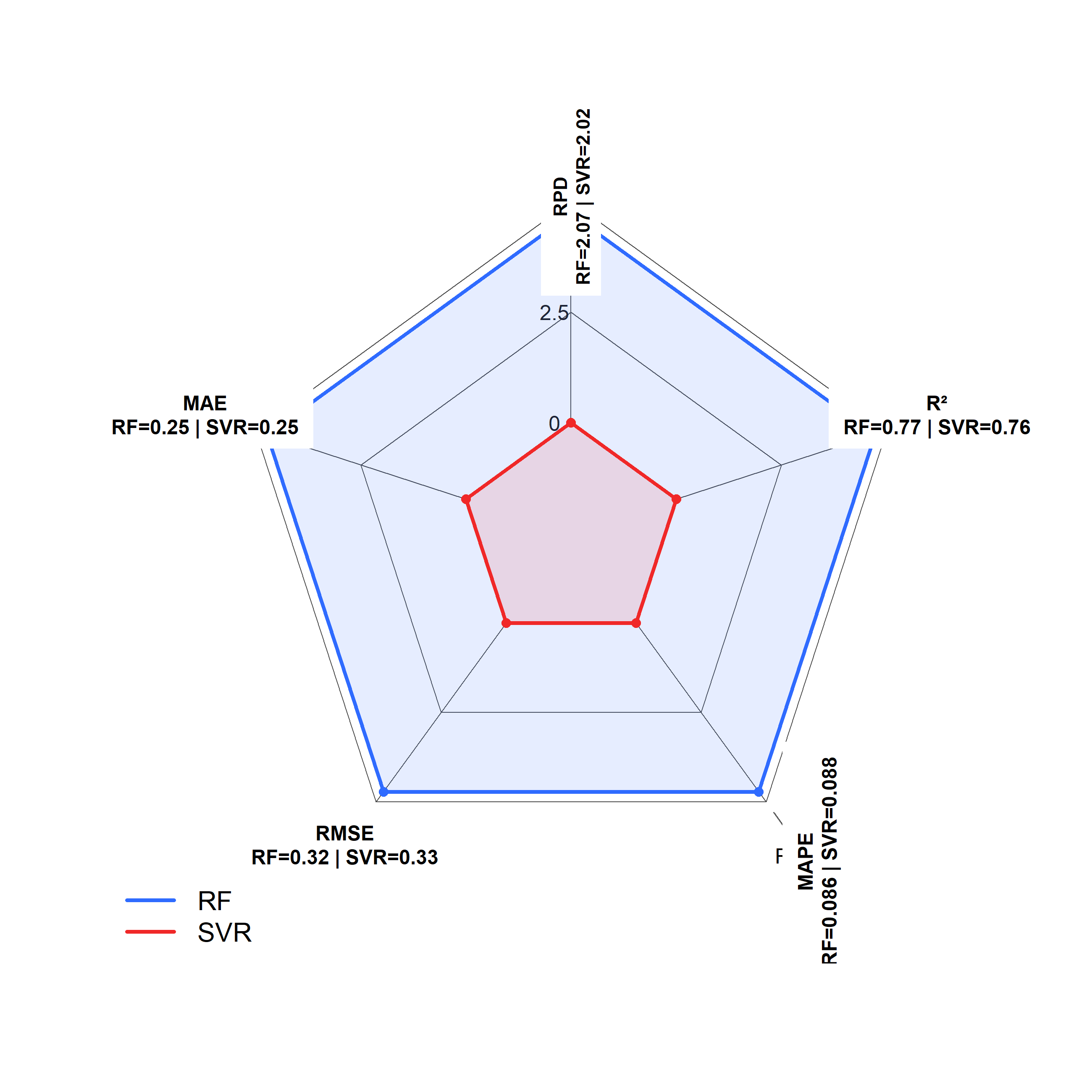

Supplement: Supplementary file 1 [file plants-15-01385-s001.zip › Figure S1 .tiff]
